# Potential Use of Remote Telesonography as a Transformational Technology in Underresourced and/or Remote Settings

**DOI:** 10.1155/2013/986160

**Published:** 2013-01-28

**Authors:** Linping Pian, Lawrence M. Gillman, Paul B. McBeth, Zhengwen Xiao, Chad G. Ball, Michael Blaivas, Douglas R. Hamilton, Andrew W. Kirkpatrick

**Affiliations:** ^1^Department of Ultrasound, The First Affiliation Hospital, Henan University of Traditional Chinese Medicine, Zhengzhou, Henan 450000, China; ^2^Department of Surgery, University of Manitoba, Winnipeg, MB, Canada R3T 2N2; ^3^Departments of Surgery and Division Critical Care Medicine, University of British Columbia, Vancouver, BC, Canada V6T 1Z4; ^4^Regional Trauma Services, Foothills Medical Centre, EG 23, Calgary, AB, Canada T2N 2T9; ^5^Department of Surgery, Foothills Medical Centre, Calgary, AB, Canada T2N 2T9; ^6^Northside Hospital-Forsyth, Cumming, GA 30041, USA; ^7^Department of Medicine, Foothills Medical Centre, Calgary, AB, Canada T2N 2T9; ^8^Department of Critical Care Medicine, Foothills Medical Centre, Calgary, AB, Canada T2N 2T9

## Abstract

Mortality and morbidity from traumatic injury are twofold higher in rural compared to urban areas. Furthermore, the greater the distance a patient resides from an organized trauma system, the greater the likelihood of an adverse outcome. Delay in timely diagnosis and treatment contributes to this penalty, regardless of whether the inherent barriers are geographic, cultural, or socioeconomic. Since ultrasound is noninvasive, cost-effective, and portable, it is becoming increasingly useful for remote/underresourced (R/UR) settings to avoid lengthy patient travel to relatively inaccessible medical centers. Ultrasonography is a user-dependent, technical skill, and many, if not most, front-line care providers will not have this advanced training. This is particularly true if care is being provided by out-of-hospital, “nontraditional” providers. The human exploration of space has forced the utilization of information technology (IT) to allow remote experts to guide distant untrained care providers in point-of-care ultrasound to diagnose and manage both acute and chronic illness or injuries. This paradigm potentially brings advanced diagnostic imaging to any medical interaction in a setting with internet connectivity. This paper summarizes the current literature surrounding the development of teleultrasound as a transformational technology and its application to underresourced settings.

## 1. Introduction

Despite revolutions and quantum leaps in the development of technology and health care systems, disadvantaged populations, separated from the mainstreams of modern technology by either geography, duty, choice, or fate, bear an increased burden of mortality and morbidity [1]. Such populations in remote/underresourced (R/UR) settings have limited access to the advanced medical health care that is the norm in the developed world. For instance, rural trauma such as the one after motor vehicle accidents may have mortality rates up to three times the national average [2, 3]. Preventable deaths in some rural areas are twice that of urban communities. While this may be explained by delays in transfer to definitive care from remote areas [3], other factors seem to come into play over and above distance alone. Often the homeless, dying within the direct line-of-sight of a major medical center, are as practically separated from potential life-saving care as a climber on Mount Everest, a soldier immobilized by enemy action, or a village dweller in the developing world.

In a vacuum of primary and tertiary care, it may be difficult to prioritize what aspects of modern medicine are most required by disadvantaged populations. The default response might be “everything.” Nonetheless, being able to accurately diagnose and assess the severity of a medical problem or condition is unquestionably a critical enabler of education, counseling, and intervention. Ultrasound represents an ideal diagnostic tool since it is noninvasive, inexpensive, highly portable, and can be utilized, in a practical manner, on-site in many R/UR settings. Ultrasound imaging has been appreciated for years by the World Health Organization (WHO). The WHO has long considered ultrasound as one of the most important technologies that developing countries need [4], rating access to general-purpose ultrasonography as a minimum global standard [5].

With current advances in ultrasound technology, coupled with less expensive hardware, portable ultrasound has become the visual “stethoscope” of the 21st century for physicians. Ultrasound machines have become smaller, more powerful, and, most importantly, cheaper, such that many health systems are increasingly able to make ultrasound a form of widely accessible health information technology (HIT). Gillman and Kirkpatrick conceptualize ultrasound as simply another dimension or “sense” to the physical exam that allows physicians to see through the skin and confirm or refute what their other senses and the clinical setting have suggested [6]. When used by those appropriately trained, ultrasound assists a range of health professionals to provide better care at the bedside, including physicians [7], nurses [8], and paramedics [9]. It also allows for expansion beyond the traditional hospital environment to a range of R/UR settings including the battlefield [10], onboard aircrafts or ships, on-site at natural disasters, and even at high altitudes during mountain expeditions [11].

However, despite the ultrasound's potential for helping almost any care provider, the generation and interpretation of US images is still very user-dependent and requires a highly skilled operator to obtain and interpret the images. As a result, even with the appropriate technology, many patients in geographically or socially isolated area may still be disadvantaged by the absence of a trained professional to operate it. Like with any diagnostic test, erroneous use of ultrasound may turn an uninformed health care provider into one whom is misinformed with potential catastrophic consequences. 

 This presents a conundrum, wherein the right diagnostic tool often exists, has demonstrated utility, but those in need of its benefits are untrained to use it. This has justified initiatives utilizing advances in information technology (IT) to allow remote nontraditional health-care providers to obtain on-site sonograms guided by distant content experts. This technique, recognized as telesonography, or tele-ultrasound (TUS), allows for interpretation of imaging exams obtained at a distance from where the interpreting ultrasonographer is located [12]. These efforts were largely initiated by the National Aeronautics and Space Administration (NASA) to allow nonphysician astronauts onboard the International Space Station to conduct ultrasound examinations with real-time guidance from terrestrial experts. Thus, in the absence of any other diagnostic imaging resources, accurate diagnosis can still be made for most of the medical conditions that might arise in low Earth's orbit [13–16]. While these initial efforts were supported by all the technical infrastructure of NASA, advances in connectivity, accessibility, and availability of hand-held computing and communication devices are making real-time mentored tele-ultrasound (RTMTUS) a service that can be brought to R/UR settings in a back pack or even a pocket and potentially used by any nontraditional care provider on any patient [17–19]. In this paper, we will highlight the development of tele-ultrasound technology and its potential to aid in the care of the most disadvantaged patients. In its seminal 2001 report, the Institute of Medicine stated that “healthcare delivery has been relatively untouched by the revolution in information technology that has been transforming nearly every other aspect of society” [20, 21], and that the absence of applying advances in information technology to improve administrative and clinical processes is disturbing [21]. We believe this previously true statement may become partially false if RTMTUS can reach its full potential. This sentiment is also in alignment with the Institute of Medicine which has stated that the use of information technologies to improve access to clinical information and support clinical decision making including using the internet to provide 24/7 responsiveness to patients needs is needed for the redesign of the 21st century health care systems [21].

## 2. The Remote Transmission and Interpretation of Ultrasound: The History of Technological Development

Ultrasound is a noninvasive medical technology that can increasingly be applied to nearly every aspect of clinical diagnosis and has unique attributes (no ionizing radiation, can be both rugged and portable, inexpensive, and generally free from harmful contrast agents) comparing favorably to most other imaging options. In recent years, the availability of inexpensive portable ultrasound machines has provided medical care providers a practical option for imaging in R/UR settings [22]. However, this technology is user dependent, and in most health-care systems, images are obtained by extensively trained and board-certified technicians and are then later interpreted by imaging experts far removed from the patients' bedside. These limitations would generally prevent widespread application of legal and medically safe ultrasound as an imaging technology for R/UR environments were it not for coincident revolutions in the adoption of point-of-care (POC) ultrasound by clinicians and the growing power and availability of communication technologies. In particular internet connectivity, voice-over-internet-protocol (VOIP) software, and hand-held computers or “Smartphones” have revolutionized individual access to potential tele-ultrasound tools. 

**Figure 1:**
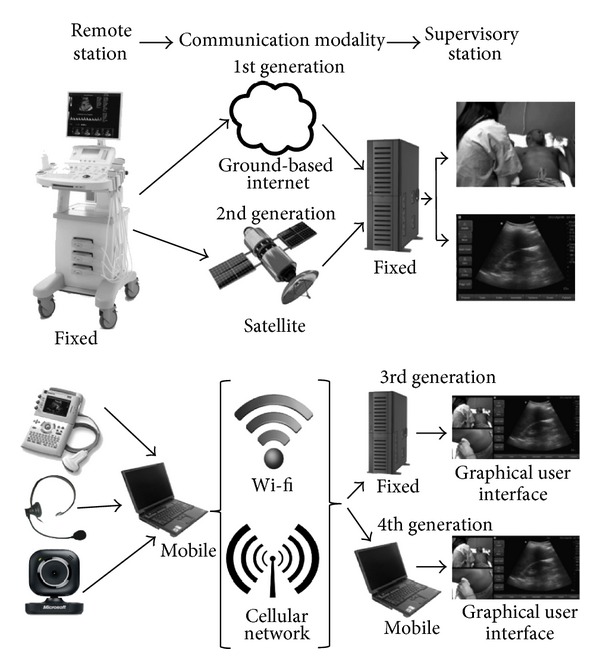
Schematic depiction and example of the system construction of the four generations of teleultrasound networks used to provide mentored tele-ultrasound initiatives.

 In the early years of tele-ultrasound initiatives it was considered a technologically demanding field, requiring high data transfer rates for the direct involvement of experts in real time relative to the internet capabilities of the time [23]. This is changing. For the ease of discussion we have considered 1st generation tele-ultrasound to comprise the original systems based on telephone line internet connections. Second (2nd) generation systems are those based on dedicated internet connections with the most geographically flexible being satellite based. We are proposing that 3rd generation systems consist of those utilizing wireless- or satellite-based internet connections to allow the remote care provider to image a patient anywhere where there is internet connectivity, whereas 4th generation tele-ultrasound systems are also wireless based and utilize both mobile receiving and transmitting stations to free the mentor from being tied to any physical location when responding to a call, in contrast to the 3rd generation which involves fixed physical locations (Figure 1). 

### 2.1. 1st Generation Teleultrasound

 The concept of using technology to enable remote care is not new and dates to the first clinical encounters over early telephones. Thus, teleradiology is a branch of telemedicine, of which tele-ultrasound is a subbranch. Gershon-Cohen and Cooley first described the clinical application of tele-radiology, which they named “telognosis” in 1950 [24]. Tele-ultrasound is a specific form of tele-radiology that enabled an expert interpreter to guide a caregiver located at a remote site through teleconferencing technology. When first described, still ultrasound images were transmitted over land-based telephone lines [25]. They noted that their requirements in dealing with possible fetal cardiac anomalies required highly accurate images, and it was important to acquire all related information about a fetus during the ultrasound examination. Considering the sensitivities of the situation and their technology, they highly recommended that real-time video transmission be applied for these consultations. The transmission of video images was also attempted, but owing to the large bandwidth requirements compared with still pictures, the quality of the images was not satisfactory for medical use [26]. Subsequently, Brebner and colleagues examined low bandwidth tele-ultrasound [27]. Even though the dynamic ultrasound images transmitted over low-bandwidth were thought to be both diagnostically acceptable and accurate, observers were not satisfied with the low-bandwidth transmission of ultrasound images at 128 kbits/s [27]. 

Chan et al. studied the transmission bandwidth requirements for accurate diagnoses when performing real time fetal tele-ultrasound consultations. The authors concluded that the majority could be diagnosed using a 384 kb/s link, which was the most cost-effective transmission rate [28]. Smith and Brebner evaluated a telemedicine service for prenatal diagnosis which carried out 85 videoconferences between a remote hospital in Elgin, Scotland, and the fetal medicine unit in Aberdeen using Integrated Services Digital Network (ISDN) transmission also at 384 kb/s and similarly concluded that the system was clinically useful [29]. 

### 2.2. Higher Generation Teleultrasound

Second generation trauma telemedicine studies have subsequently taken advantage of the developing technology and internet connectivity. Investigators have extended this early work using custom, internet-based videoconferencing software to provide high quality, real-time video links between tertiary care facilities and more remote settings [30–32]. However, standard videoconferencing equipment can be expensive and may impose large start-up costs leading many to consider cost to be the main barrier to the widespread application and adoption of telemedicine.

However, since both these generations of pioneering studies, IT networks have expanded dramatically in bandwidth, global penetrance, and accessibility to service the hand-held computer or smartphone. Developments in IT, thus, continue to revolutionize connectivity thus reducing costs and logistic requirements. Investigators at the University of Calgary have demonstrated worldwide tele-ultrasound connectivity with VOIP software allowing voice and other multimedia (e.g., video) to be transmitted via the internet. This use of the internet in place of the public-switched telephone network (PSTN; normal telephone) potentially makes tele-ultrasound available in any R/UR setting in which a smartphone has connectivity. The particular system constructed in Calgary utilized a freely accessible internet service (Skype) that currently provides low cost videoconferencing services to millions of people and businesses worldwide [23]. Using such a system, McBeth et al. conducted a series of trials with the assistance of healthy volunteers at a variety of remote sites to demonstrate the feasibility and applicability of these techniques conducted primarily over a smartphone [18, 19]. Other investigators have also utilized a variety of commercial video-streaming devices to transmit real-time streaming ultrasound images over either the internet [33] or satellite transmission [34, 35], with potential remote guidance potentially conducted by telephone or without a video camera depiction of the user [34, 35].

## 3. Principles of Real-Time Mentored Tele-Ultrasound

Although formal controlled studies are lacking, we believe that the enabling concepts in order to achieving the most accurate diagnoses include excellent communications, strict but simple protocols, and if possible, short-time training courses. In the initial tele-ultrasound efforts, the lack of direct interaction between those performing and interpreting prevented any real mentoring or correction at the time of imaging. For instance, store-and-send systems only allow electronic storage of still images and video clips for future reference. Thus, if any critical diagnostic information was missing, the interpretation would be incomplete or inaccurate without the chance to correct until long after the attempted interpretation. However, with the development of technology allowing simultaneous display of the examiner, patient, and transducer positions, real-time feedback to produce the desired images becomes immediate. For the local providers, becoming familiar with telesonography equipment and learning ultrasound basics and scanning techniques greatly help to provide experts with the images necessary for accurate diagnoses and reduce the time and effort necessary. The initial studies conducted from the International Space Station (ISS) involved briefing the astronauts months before the mission and thereafter with [13, 14], and without [15], a prestudy refresher modules and cue cards to assist the astronauts inflight. Without being able to especially visualize the on-site examiners hands, the NASA investigators made extensive use and reference to the cue cards which provided standard terminology and facilitated accurate communications [15]. 

Although adequate connectivity is critical, the most important aspect of communications relates to the human interaction between the mentor and mentee. Although the technique was created to serve the needs of highly educated astronauts, RMTMUS techniques seem to be easily applied to much less formally trained providers including nurse practitioners [36], ski patrollers and paramedics [9] (Kirkpatrick AW, personal conversation), and even children [37]. The key enabler appears to be simply the willingness of the remote on-site provider to listen, pay attention, and to respond to the direction of the remote expert, implying that any willing remote responder who understands what the remote mentor is requesting could attempt this procedure if the situation called for it. 

## 4. Demonstrated Applications in Underresourced and Remote Settings

Underresourced settings in the Western world are associated with poor, inner-city minority communities, rural residents, and other vulnerable populations [38]. It is these populations that have been shown to be the last to benefit from medical innovation [39]. Whether vulnerable by choice, circumstance, or legal sanction, these patients share common characteristics with those in developing countries or astronauts in Earth's orbit as they may be geographically isolated in terms of health care delivery and patient retrieval to higher level medical care when necessary. 

### 4.1. Space Medicine Applications

 Space exploration represents an interesting and extreme example of a remote environment where “underresourced” becomes a relative term. More specifically, the array of HIT and DI technologies that are normal in advanced Western hospitals will not be available. However, some of the most valued of these may be selected and brought along on the mission. Respecting a complex decision-making matrix of portability, power-consumption, mass, side-effects, and utility, ultrasound is that singular DI modality selected for space. Currently, and into the predictable future, ultrasound is the only medical imaging modality available onboard the International Space Station (ISS) [40–42]. Studies have concluded that such ultrasound systems are capable of high-definition sonographic imaging of many if not most organ system and their common disease states may be obtained using inexperienced astronauts who obtain remote guidance from terrestrial experts in the Mission Control Center on Earth [43, 44]. 

In the inaugural article, Sargsyan et al. reported that terrestrial experts conducted a remotely guided Focused Assessment with Sonography for Trauma (FAST examination) with excellent clinical results and speed using private real-time two-way audio and a private space-to-ground video downlink [15]. Further efforts thereafter concluded that nonphysicians with minimal training could generate complex, diagnostic-quality complex examination images regarding musculoskeletal, retroperitoneum, and pelvis examinations when directed by a remote-based expert on International Space Station [16, 44, 45]. This same team evaluated the ability of nonphysician crewmembers to obtain diagnostic-quality musculoskeletal ultrasonographic (US) data of the shoulder by following a just-in-time training algorithm and using real-time remote guidance [13]. Further, a comprehensive, high quality ultrasound examination of the eye was performed with a multipurpose imager aboard the ISS by a nonexpert operator using remote guidance [14]. Finally, with preparation and planning, increasingly complex examinations have been addressed. The efficacy of remotely guided teleechocardiography was evaluated by Hamilton et al. through a prospective trial of echocardiography conducted on six crew members onboard the ISS [46]. 

### 4.2. Terrestrial Applications

 Teleultrasound is also increasingly being studied in terrestrial environments. This has allowed telementored examinations to be studied between different locations within the same hospital [9] and between sites including rural referring centres and urban trauma centres [47, 48]. For instance, a 2nd generation telemedicine link was created between Banff and Calgary allowing bidirectional videoconferencing and unidirectional US transmission. In this study, critical anatomic characteristics of the FAST exam were identified in over 98% of all examinations that were attempted [47]. 

Telementored examinations have also been studied in the field utilizing 3rd and 4th generation cellular, wireless, and satellite communications [12, 49]. This has allowed for on-site assessment and triage during mass casualty events or at military field triage sites [50]. The potential capabilities of tele-ultrasound have been demonstrated not only pretransport but also in transit both onboard a small airplane in flight [12] as well as at sea on a merchant ship at sea [51]. Otto et al. successfully used a satellite telemedical connection with a remote expert to guide a thoracic ultrasound examination at an Advanced Base Camp on Mount Everest thus demonstrating that tele-ultrasound with remote expert guidance can provide robust diagnostic capabilities even in the most austere locations [52]. Telemedicine services have also been used over greater distances both between different cities of the same country [29, 53–55] as well as between countries [18, 19]. Telementored telesonography has even been used to view real-time ultrasound images across continents. In one study, remote experts in Aberdeen, Scotland, were able to easily view an exam performed in Calgary, Alberta. The examiner's hands and probe placement, in addition to the resultant ultrasound images and bidirectional communication, were all provided by transmission over a smartphone with the images deemed to be of diagnostic quality and in real time [18, 19]. Similar studies have been performed between remote centres in Honduras and the United States [56] as well as across the Atlantic Ocean [57]. 

### 4.3. Remote Telesonography in Clinical Application

*Trauma.* Trauma represents one of the leading causes of preventable death worldwide. The initial phase of trauma resuscitation, often referred to as the “golden hour of trauma,” relies heavily on the early identification of life threatening injuries. Ultrasonography represents a noninvasive technology that can increasingly be applied to almost every aspect of trauma diagnosis. As mentioned earlier, as the technology continues to develop, portable ultrasound units within the emergency department have allowed ultrasound to transform from a confirmatory diagnostic test to an extension of the physical examination [6, 22]. 

Tele-ultrasound has the potential to expand this application beyond the realm of trauma. This includes any medical centre and any prehospital location with internet connectivity. In this way, life threatening and immediately treatable injuries can be identified and expediently dealt with. For instance, emergent diseases such as pneumothoraces can be excluded immediately by a remote expert that can be economically linked to almost any responder over cellular networks, fractures can be anatomically reduced with alignment confirmed, and asystole confirmed preventing unnecessary resuscitative efforts. Additional information, even if it cannot be immediately acted upon, can have important triage implications. For instance, positive FAST examinations may alter triage protocols in a variety of trauma patients. 

Strode et al. initiated studies of infield FAST examinations on a healthy volunteer transferred wirelessly at distances of 1,000 and 1,500 feet using a vest-mounted microwave transmitter redirected over satellite to a remote hospital for review by emergency physicians and a radiologist. Image quality and interpretability at these distances were not degraded compared with on-site images [49]. More recently Boniface et al. examined the capability of ultrasound-naïve paramedics to obtain interpretable Focused Assessment with Sonography for Trauma (FAST) images under the remote direction of emergency physicians (EPs). Fifty-one paramedics performed their first FAST examinations and were able to successfully complete 100% of the views of the FAST [9]. In a separate study, an internet link was created to allow bidirectional videoconferencing and unidirectional US transmission between the Banff Springs Hospital and the referral trauma facility in Calgary, Alberta. Investigators directed an extended FAST examination (EFAST) utilizing NASA algorithms and concluded that remote real-time guidance of an EFAST using telesonography appears feasible [47, 48]. A positive tele-FAST in this setting directed one unstable patient away from the ER, instead, justifying a direct operating room resuscitation. Ongoing efforts in this project relate to efforts to increase the responsiveness of the responding mentors through the use of mobile computing devices upon which to view the Banff ultrasound images.

### 4.4. Obstetrical Applications

 Ultrasound is inherently well suited for the assessment of fetal problems because of a lack of fetal exposure to ionizing radiation. It remains an indispensable tool for the confirmation of intrauterine pregnancy, monitoring of fetal growth, and evaluation of pregnancy-related complications. Further, when a fetal anomaly is suspected, accurate diagnosis is essential before informing and discussing with parents. However, geographically isolated pregnant patients often have limited access to tertiary fetal diagnostic centers including ultrasound services. Thus, telemedicine is an obvious diagnostic tool to provide remote communities with accurate prenatal screening while attempting to avoid the risks of unnecessary travel [58], especially given the risks to patients and crew in both ground and aeromedical transport [59, 60]. Teleultrasound has already allowed remote diagnostic consultations with specialists at tertiary care prenatal ultrasonography centers [61]. A program based in Australia has created such a telemedicine link between remote areas and tertiary care prenatal ultrasonography centers [62, 63]. Three-dimensional ultrasound is increasingly being used in fetal diagnostics. Three-dimensional ultrasound datasets can also be stored in the remote unit and then transmitted to a consultant for more complex retrospective analysis [64]. 

Until recently, the high costs of medical equipment and telecommunication networks have limited the application of telemedicine. Now, with markedly reduced costs of equipment and the ubiquitous connectivity of the internet, tertiary real-time fetal tele-ultrasound consultations are accurate, clinically useful, and cost effective [65]. Some concerns remain about limitation of required bandwidth since the transmission of real-time fetal ultrasound images involves higher than normal volumes of data [66]. However, in blinded studies using various communication technologies and varying bandwidths [67], these concerns are not supported [68]. Ferlin et al. evaluated the diagnostic quality of first-trimester ultrasound images transmitted in real time using low-cost telecommunications and concluded that the quality of images was suitable for screening regarding chromosomal abnormalities [69]. 

### 4.5. Cardiology

 Since the introduction of echocardiography by Edler and Hertz in 1953, it has become an indispensable tool in clinical decision making [70]. The small size of handheld devices allows professionals to carry an ultrasound scanner portably and to use it conveniently almost anytime and anywhere during routine clinical work. Previous investigators have documented that trained primary care practitioners or nurses can perform cardiac ultrasound exams on neonates at risk for heart disease with telemedicine supervision [71]. Recent studies have evaluated the feasibility, accuracy, and user acceptability of performing remote fetal echocardiography in different tertiary care centers with expertise in 4D echocardiography. Prenatal congenital heart disease has been diagnosed using four-dimensional spatio-temporal image correlation (STIC) telemedicine via Internet [54, 72, 73]. McCrossan et al. evaluated the feasibility, accuracy, and user acceptability of performing remote fetal echocardiograms (FEs) and overall telelink quality. Eleven out of 12 components of the FE were confidently assessed. The authors concluded that congenital heart disease could be confidently diagnosed and excluded by remote FE. It is also noted that this application of telemedicine could improve access to fetal cardiology and support radiographers screening for congenital heart disease [55]. 

With newer 3rd and 4th generation smartphone-based applications, remote expert echocardiographic interpretation can provide backup support to point-of-care diagnosis by nonexperts. Mobile-to-mobile consultation may thus improve access in previously inaccessible locations [56]. Lim et al. assessed the feasibility of using a camcorder mobile phone for evaluation of left ventricular (LV) systolic function with cardiac echocardiography in emergency patients. The measurements of LV ejection fraction based on the transmitted video displayed on a mobile phone were comparable with the original video displayed on the LCD monitor of the ultrasound machine [74]. Echocardiography was conducted on crew members onboard the ISS by Hamilton et al. Investigators evaluated the efficacy of remotely guided teleechocardiography, utilizing just-in-time e-training modules to determine what is “space normal” echocardiographic data, via video downlink and two-way voice communication [46].

### 4.6. Musculoskeletal Applications

 Musculoskeletal injuries are common, and ultrasound is again an ideal imaging diagnostic modality for this application in remote areas because of its portability and low cost. Many bony and soft tissue injuries are well seen with ultrasound [75]. Nowak et al. have recently reviewed its use for real time, mountainside diagnoses of these musculoskeletal injuries [76]. A teleultrasound connection with remote guidance of nonmedical, inexperienced ultrasound subjects from the Arctic Circle recently obtained useful musculoskeletal images from a portable ultrasound system [34]. Imaging of groin, knee, ankle, elbow, and shoulder has also been performed on board the ISS by nonphysician crew members and all real-time US video stream, and still captured images were considered adequate for diagnostic interpretation by terrestrial experts [43, 45]. 

### 4.7. Future Directions

 With the continuous development of the internet and other information technologies, tele-ultrasound represents an ideal clinical tool in underresourced settings because of the portability of equipment and the noninvasive, rapid, versatile, and repeatable examination. In the future, its use in austere locations will continue to expand; onboard the International Space Station, on the battlefield, on remote islands, and in other under resourced areas. Further interventional procedures including tele-ultrasound guided acute procedures such as thoracocentesis, pericardiocentesis, or paracentesis will likely empower on-scene care in R/UR settings.

In recent years, many tele-echography robotic systems have been developed. With this technology, the physician can guide the motion of the transducer by the paramedic from a remote location. Ito et al. developed a tele-echography (FAST) robot system that was tested by a paramedic for the assessment of trauma patients both in ambulance and at the injury scene [77]. Even though these systems are currently used primarily on a research basis, considering the speed of technological developments, we are sure that these techniques and telecommunications linkups will become much more popular and useful in R/UR settings. The merger of the high resolution smart phones and ultrasound devices has made steady progress, and in the future, these common technologies may be the primary means of providing remote guidance capability to support diagnostic and therapeutic imaging anywhere on earth.

## 5. Conclusions

 Compared with other diagnostic imaging tools, teleultrasound represents an ideal imaging modality for patients in underresourced areas. With advancements in telecommunication systems, the practice of remote telesonography has the potential to play a vital role in raising the quality of health care in underresourced setting both in space and on the ground. At the present time, the technology appears to be ready, but it has not been proven effective in routine clinical use, and this constitutes a target for future researchers and clinicians. Ultimately, the potential benefit to patients could be the delivery of high quality diagnostic capabilities in almost all locations at any time. With continued improvements in technology and dissemination of education and user confidence, the future is bright.
